# The role of the gut-brain axis in depression and anxiety disorders

**DOI:** 10.1192/j.eurpsy.2021.1840

**Published:** 2021-08-13

**Authors:** M. Trigo

**Affiliations:** Psychiatry, Centro Hospitalar Universitário do Algarve, Faro, Portugal

**Keywords:** Gut-brain axis, Depression, anxiety disorders, microbiota

## Abstract

**Introduction:**

There is a bi-directional biochemical communication pathway between the gastrointestinal tract and the central nervous system, referred to as the “gut–brain axis”. Studies show that bacteria in the gastrointestinal tract, including commensal, probiotic, or pathogenic, can affect brain’s function. Since there is a symbiotic relationship between gut microbiota and the brain, changes in its composition can lead to dysbiosis, which plays a role in many psychiatric disorders, such as depression and anxiety, and therefore becomes a potential therapeutic target.

**Objectives:**

To examine data from recent studies regarding the gut-brain axis and its relationship with psychiatric disorders, such as depression and anxiety.

**Methods:**

Review of the most recent literature regarding the gut-brain axis and its relationship with depression and anxiety disorders. The research was carried out through the MedLine, PubMed, UptoDate, ScienceDirect, SciELO and SpringerLink databases, using the terms “gut-brain axis”, “depression” and “anxiety”, until December 2020.

**Results:**

There is a relationship between dysbiosis of microbiota and some psychiatric disorders, particularly depression. Symbiosis may be restored by purposefully manipulate the gut microbiota using therapies such as probiotics, therefore enhancing beneficial bacteria in the gastrointestinal tract and improving symptoms of depression.

**Conclusions:**

Although probiotics can be used in the treatment of depression, further research is needed in order to carefully determine parameters such as the duration of treatment, dosage and drug interactions. Nonetheless, a better understanding of the gut-brain axis may arise new approaches on how we prevent and treat mental illnesses.

**Disclosure:**

No significant relationships.

